# Nanostructured Channel for Improving Emission Efficiency of Hybrid Light-Emitting Field-Effect Transistors

**DOI:** 10.1021/acsphotonics.3c01080

**Published:** 2023-12-10

**Authors:** Alejandro Galán-González, Piotr Pander, Roderick C. I. MacKenzie, Leon Bowen, Dagou A. Zeze, Robert J. Borthwick, Richard L. Thompson, Fernando B. Dias, Mujeeb Ullah Chaudhry

**Affiliations:** † Department of Engineering, Durham University, Durham DH1 3LE, United Kingdom; ‡ Instituto de Carboquímica (ICB-CSIC), C/ Miguel Luesma Castán 4, 50018 Zaragoza, Spain; § Faculty of Chemistry, Silesian University of Technology, Strzody 9, 44-100 Gliwice, Poland; ∥ Centre for Organic and Nanohybrid Electronics, Silesian University of Technology, Konarskiego 22B, 44-100 Gliwice, Poland; ⊥ Department of Physics, Durham University, Durham DH1 3LE, United Kingdom; # Department of Chemistry, Durham University, Durham DH1 3LE, United Kingdom

**Keywords:** ZnO, nanowires, light outcoupling, light-emitting transistors, contact resistance

## Abstract

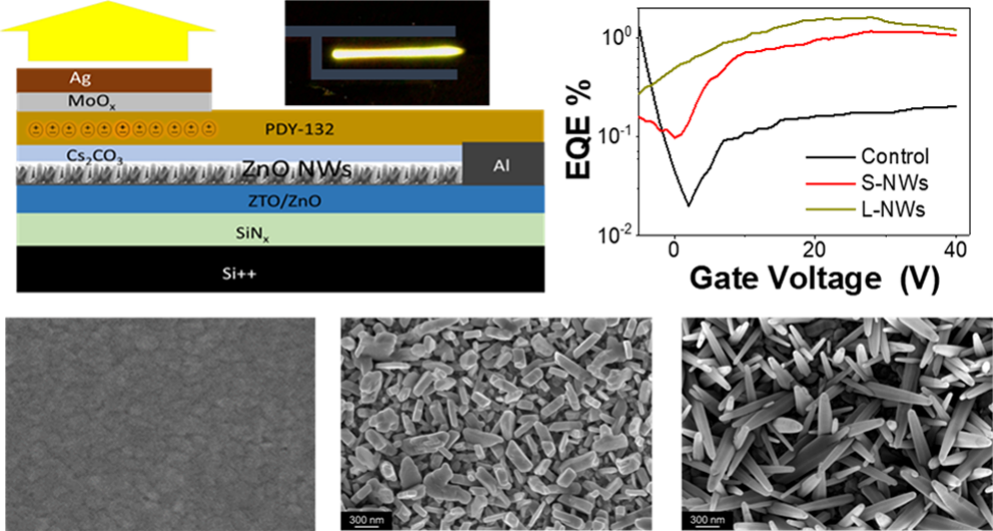

We report on the mechanism of enhancing the luminance and external quantum efficiency (EQE) by developing nanostructured channels in hybrid (organic/inorganic) light-emitting transistors (HLETs) that combine a solution-processed oxide and a polymer heterostructure. The heterostructure comprised two parts: (i) the zinc tin oxide/zinc oxide (ZTO/ZnO), with and without ZnO nanowires (NWs) grown on the top of the ZTO/ZnO stack, as the charge transport layer and (ii) a polymer Super Yellow (SY, also known as PDY-132) layer as the light-emitting layer. Device characterization shows that using NWs significantly improves luminance and EQE (≈1.1% @ 5000 cd m^–2^) compared to previously reported similar HLET devices that show EQE < 1%. The size and shape of the NWs were controlled through solution concentration and growth time, which also render NWs to have higher crystallinity. Notably, the size of the NWs was found to provide higher escape efficiency for emitted photons while offering lower contact resistance for charge injection, which resulted in the improved optical performance of HLETs. These results represent a significant step forward in enabling efficient and all-solution-processed HLET technology for lighting and display applications.

## Introduction

The combination of inorganic charge transport materials (usually metal oxide) interfacing with an emissive organic material in a hybrid light-emitting field-effect transistor (HLETs) structure provides access to a fast and relatively stable light-emitting transistor technology.^1−7^ Despite the tremendous progress that has been achieved by using hybrid material combinations and interface engineering, improvements in power efficiency are still needed to realize the full potential of the HLET technology.^5−13^ Such requirements are even more pertinent for solution-based fabrication processes that will have an impact on the display sector.^5,11−13^ As for display technology, HLETs offer an alternative pixelation design by combining the switching function and light emission into a single device.^5,11^ This multifunctionality of electroluminescence and switching in HLETs simplifies the pixel architectures.

ZnO has emerged as one of the most attractive metal oxide semiconductors owing to its abundance, low cost, and fast charge transfer dynamics.^14−16^ Moreover, the ease of its fabrication has enabled the preparation of a wide variety of morphologies that have been employed as photoanodes,^16,17^ sensors,^18,19^ and optoelectronic devices.^20−22^ Among these morphologies, nanowires (NWs) are advantageous to other ZnO morphologies or thin films as they have excellent wettability and provide a much larger surface area and shorter lateral transfer lengths thanks to their high aspect ratio while maintaining a single crystalline quality.^23,24^ ZnO NWs are small 3D nanostructures that can provide a large surface area to enhance the charge injection and photonic structure for increasing the outcoupling efficiency of the light. All of these features make ZnO NWs an ideal charge injector that can efficiently connect any active layer with an organic emissive layer, boosting the optoelectronic performance of solution-processed HLETs.

Herein, we demonstrate efficient, multilayer, and solution-processed HLETs with a nanostructured metal oxide channel for charge-transporting and a polymer-based emissive layer. The HLETs exhibited excellent electron mobility in the range of 1–7 cm^2^ V^–1^ s^–1^, mainly due to the presence of the metal oxide charge transport layers (ZTO and ZnO). Integration of NWs led to a reduction in the ON/OFF current ratio of the HLET but resulted in higher external quantum efficiency (EQE) and luminance. Carefully engineered HLETs with longer NWs embedded in their channel exhibited a luminance of >2000 cd m^–2^ and an EQE of ∼1.18%, which is higher than any reported HLETs.

## Results and Discussion

**Figure 1:**
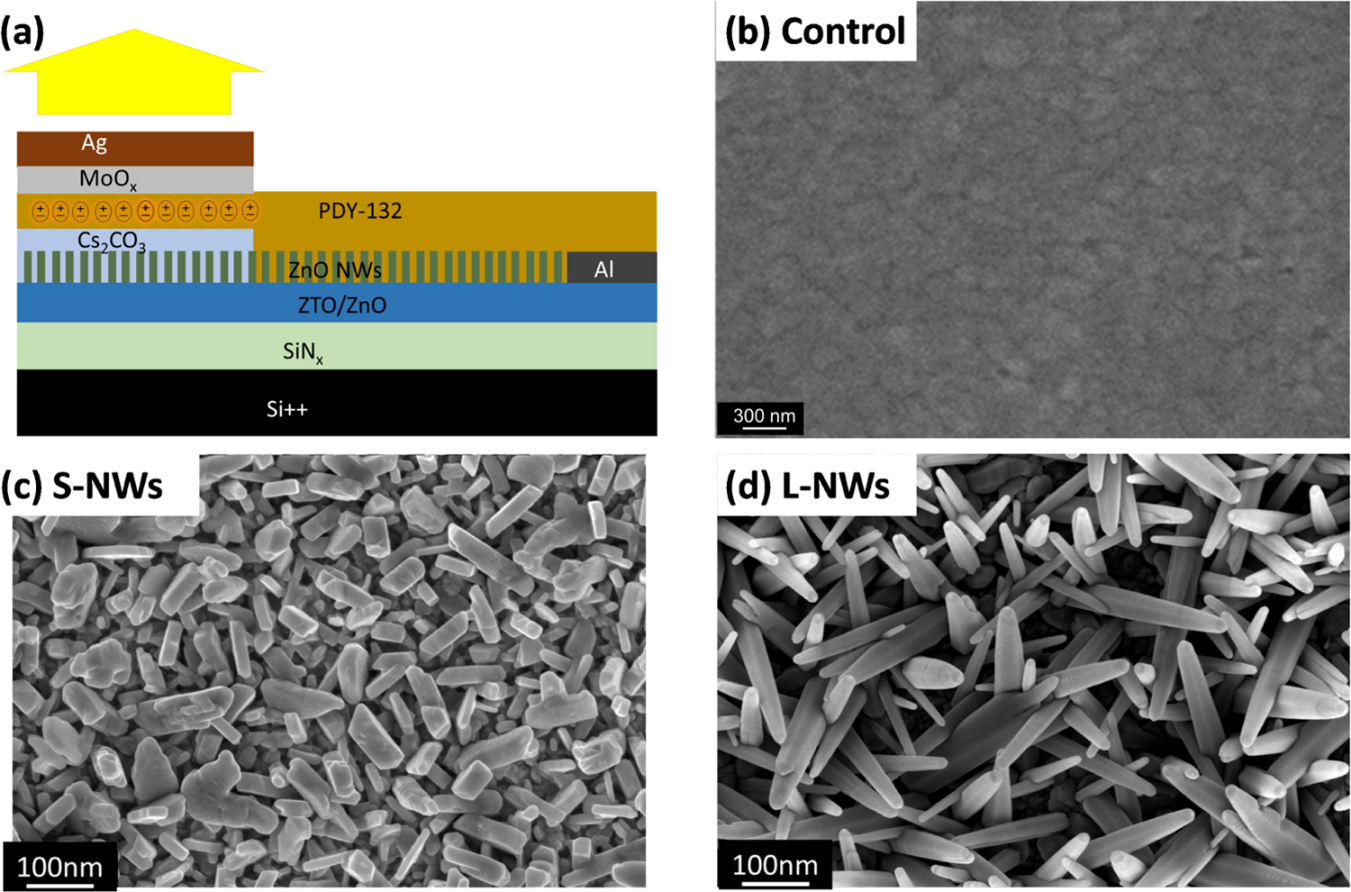
(a) Schematic of the HLET device structure. SEM micrographs of the (b) Control, (c) Short, and (d) Long ZnO nanowires (Control, S-NWs and L-NWs, respectively) that cap the active layer.

Figure 1a shows a schematic of the nonplanar electrode structure of the HLETs used in this study. Super Yellow (SY, see Figure S1a) was chosen as the emissive and hole transport layer on top of the charge-transporting layer formed by a ZTO/ZnO stack (see Figure S1b,c). The control sample consisted of the underlying ZTO/ZnO active layer with no ZnO NWs grown on top. These samples were used as the comparative basis to assess the enhancement provided by the ZnO NWs. Two sets of devices were then fabricated by growing the short nanowires (S-NWs) and long nanowires (L-NWs) on top of ZTO/ZnO. Both sets of samples showed two distinct ZnO NW growth, which is detailed in the Experimental Section. The scanning electron microscopy (SEM) images of the oxide layer are provided in Figure 1b–d for control, S-NWs, and L-NW samples, respectively. The goal of having two different NW lengths was to determine the impact that this key NW parameter could have on the device performance. The S-NWs (Figure 1c) were grown by using a shorter synthesis time and a lower reagent concentration. This sample displays a rod-like morphology, and the NWs are randomly distributed throughout the ZTO/ZnO layer with a heterogeneous size distribution.

The sample containing L-NWs is shown in (Figure 1d). The L-NWs appear to have a more homogeneous distribution and a tapered tip and nanowire-like morphology. Their increased aspect ratio is likely to considerably benefit their charge injection capabilities. In both cases, the as-grown NWs were single crystals and exhibited a wurtzite crystalline phase (see HRTEM in Figure S2).

**Figure 2:**
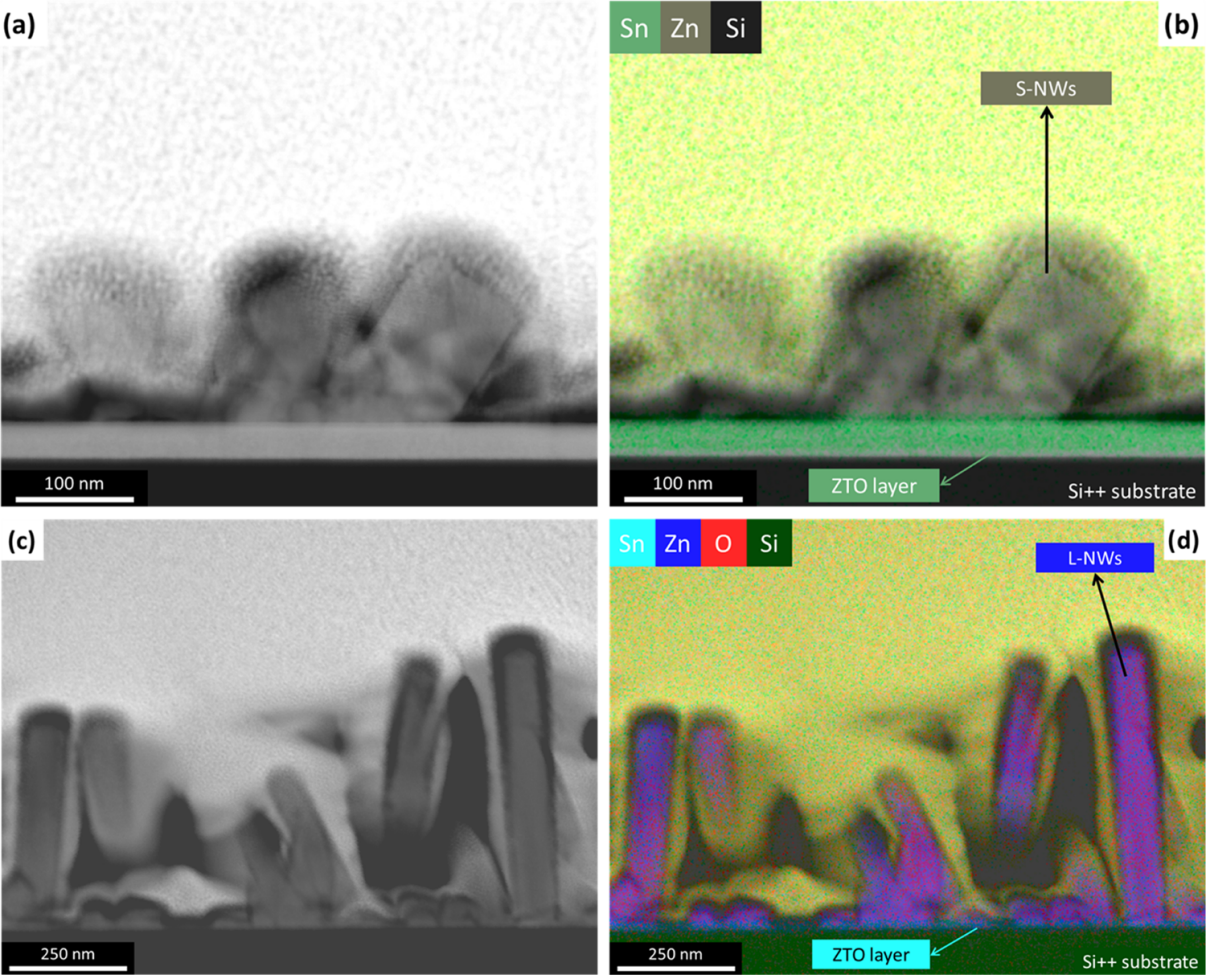
TEM micrographs and STEM mapping of the (a, b) short and (c, d) long ZnO nanowires that cap the active layer, respectively.

To better understand the morphological characteristics of the ZnO NWs, TEM and STEM analyses were carried out on both S-NWs (Figure 2a,b) and on L-NWs (Figure 2c,d), respectively. The TEM micrographs demonstrate good agreement with the expected length of the NWs, with the S-NWs being significantly shorter and wider than the L-NWs. In addition, the L-NWs are more homogeneous in both size and shape, which could yield a more intimate contact with the Cs_2_CO_3_ and the emissive layer. Given that the ZnO NWs were grown on a ZTO/ZnO stack, we consider the possibility of Sn migration from ZTO to the NWs during the hydrothermal growth and subsequent processing of the electric contacts and the emissive layer. For this reason, elemental mapping of the ZnO NWs was performed (Figure 2b,d) showing no signs of Sn migration from ZTO to the NWs. Sn appears to be constrained to the region of the ZTO layer (see Figure S3), which has a thickness of ∼35 nm, and is not observed in the ZnO nanowires. Moreover, the Zn signal is mostly limited to the as-grown NWs, possibly indicating a lower Zn content in the ZTO active layer. Weaker Zn signals can be observed closer to the substrate for L-NWs (Figure 2d). These are ascribed to the initial growth particles that form the base of the ZnO NWs and are unrelated to the ZTO active layer. The length of the S-NWs is ∼140 nm and that of the L-NWs is ∼400 nm, while the thickness of the SY layer was such that it covered the full ZTO/ZnO NW stack.

**Figure 3:**
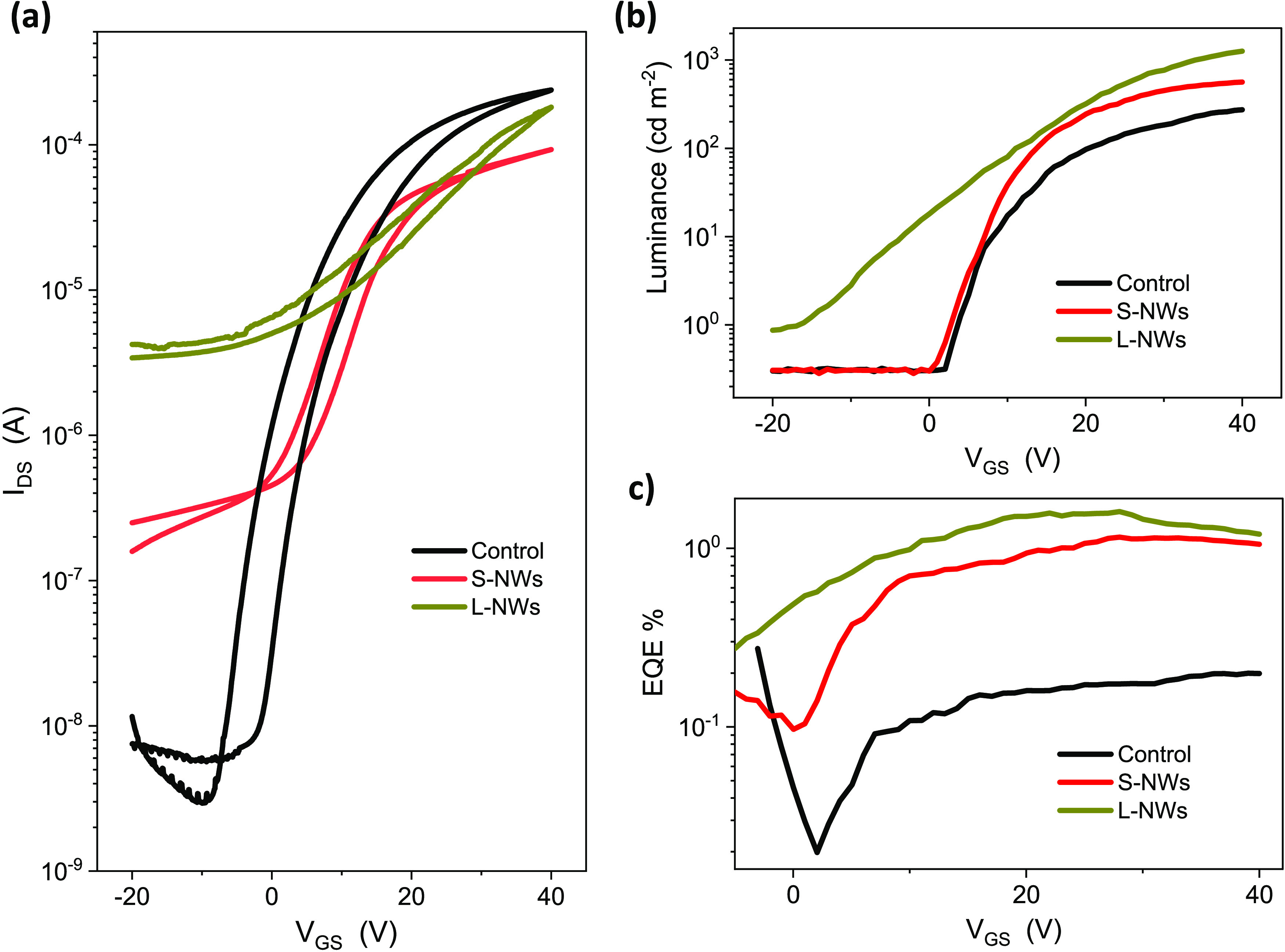
(a) Electrical and (b) optical transfer characteristics of three HLETs in the saturation regime (*V*_DS_ = 40 V). (c) External quantum efficiency comparison of HLETs in three structures.

Figure 3a shows the electrical transfer characteristics of three types of HLETs: (i) Control, (ii) S-NW, and (iii) L-NW HLETs. A higher electrical ON/OFF ratio of >10^5^ is typically observed for the control HLET. However, this ON/OFF ratio changes drastically with the incorporation of NWs in the channel i.e., S-NW HLETs exhibit ON/OFF of ∼6 × 10^2^ and, for L-NW HLETs this ratio decreases further down to ∼60. We attribute this lower ON/OFF ratio to the higher number of free carriers in NW devices available due to (i) the higher crystallinity of the ZnO NWs, and (ii) the fact that Sn concentration increases in the bottom ZTO layer as ZnO is employed by the NWs to grow from the solution and the underneath layer.^25,26^ This results in an Sn-rich underneath (ZTO) layer (with higher conductivity) that pushes the off current to higher values. Therefore, tuning the Sn concentration might help to improve the on/off ratios. The introduction of the ZnO NWs on top of the ZTO/ZnO stack results in a slightly lower electrical performance of the transistors as *I*_DS_ is decreased, i.e., similar charge carrier mobility and a much lower ON/OFF ratio. All devices showed lower gate leakage current (see Figure S4) and some degree of hysteresis in drain current when the gate bias was scanned forward and backward, and we associate this with the interfacial traps as reported.^27^ The output curves of all devices are provided in Figure S5. We observe considerable improvement in electroluminescent transfer properties of the HLETs (Figure 3b,c) upon introducing the ZnO NWs. In this regard, the luminance of the HLETs increases by 50% when the S-NWs are used. An increase by nearly 1 order of magnitude relative to the control device (∼10^3^ cd m^–2^) is observed when the L-NWs are grown on the ZTO/ZnO layer. More importantly, we observe a higher luminance of S-NW and L-NW-based devices despite lower *I*_DS_, indicating an increase in the EQE. The EQE of the HLETs is presented in Figure 3c. EQE peaks around 25 V and then reduces at higher current densities at increased gate bias. The introduction of the NWs leads to an EQE increase by nearly 1 order of magnitude relative to the control device. However, no statistically significant difference in EQE can be observed between the S-NWs and L-NWs, although the latter still provides a slightly improved EQE. These results are summarized in Table 1.

**Table 1:** Summary of the Electrical and Electroluminescent Characteristics of HLETs^a^

device parameters	control	S-NWs	L-NWs
μ_e_ [cm^2^ V^–1^ s^–1^]	0.6 ± 0.1	0.24 ± 0.04	0.5 ± 0.1
on/off	∼10^4^	∼6 × 10^2^	∼60
maximum luminance [cd m^–2^]	280 ± 20	550 ± 50	1250 ± 50
EQE at maximum luminance [%]	0.2 ± 0.05	1 ± 0.1	1.2 ± 0.1

a Statistics were taken for at least eight devices in each category. For comparison with reported HLETs, please see Table S1.

We attribute the significant enhancement of the luminance and EQE of the HLETs using ZnO NWs to the improved (i) charge injection and (ii) light outcoupling provided by the nanostructured ZnO. For improved charged injection, the NWs offer a larger contact area with the Cs_2_CO_3_ or SY layers due to the 3-dimensional structure of the NWs when compared with the ZTO/ZnO film. This enhanced interaction due to a larger area on NWs yields a better charge injection to the SY and, thus, a better device performance.

**Figure 4:**
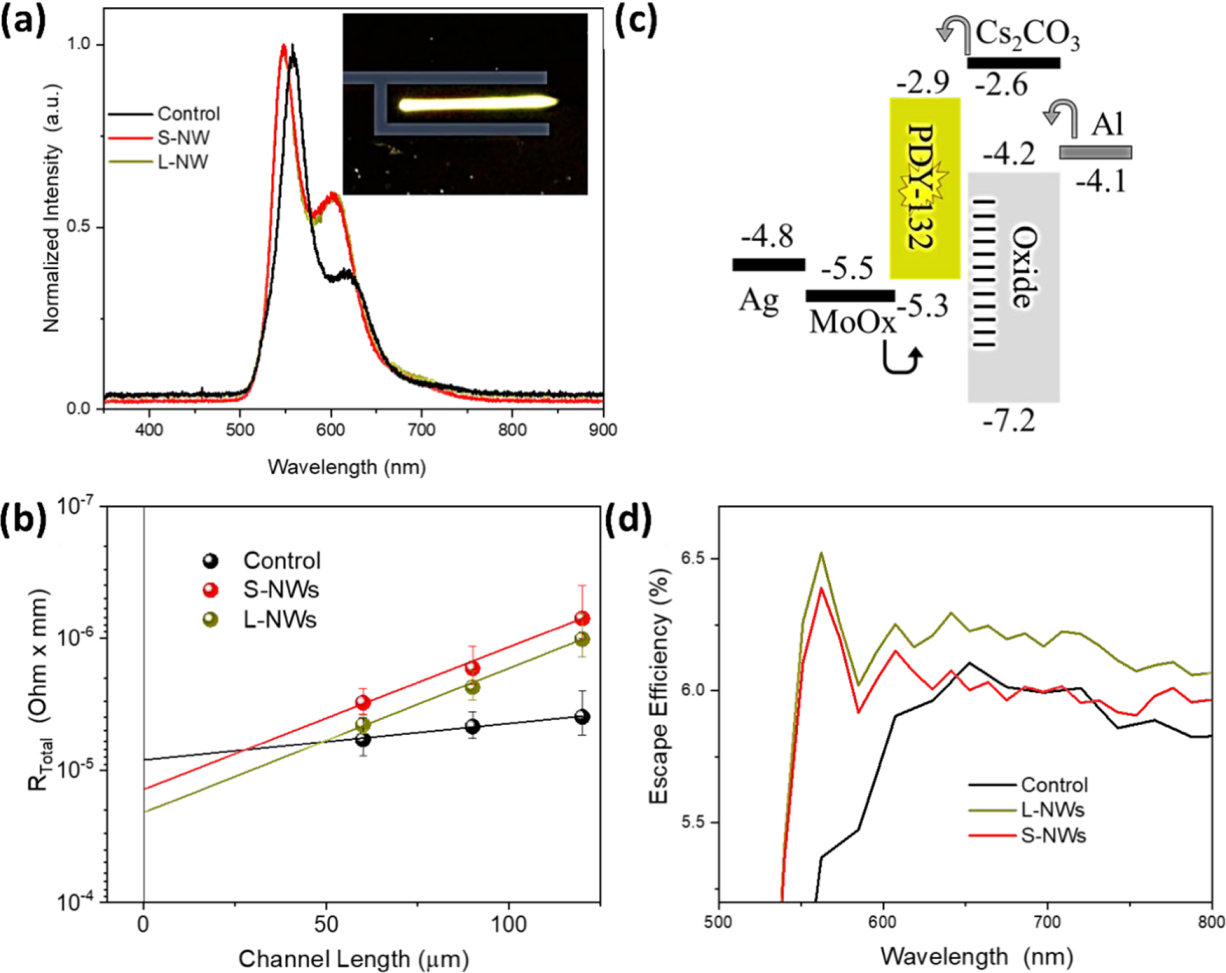
(a) Electroluminescence spectra of the three HLETs, with an inset showing a photograph of the emission from the actual HLET in operation. (b) Energy diagram of the HLETs. (c) Comparison of contact resistance of the three devices. (d) Comparison of escape efficiency of the three devices.

Figure 4a shows the EL spectra of the HLETs with the λ_EL_ = 550 nm, in agreement with the expected luminescence from SY. The inset of Figure 4a shows a digital micrograph of the actual HLET in operation, where emission can be observed coming through the drain electrode of size 0.1 mm × 2 mm. A micrograph of variable channel HLETs is provided in Figure S6. We observed the EL through the MoO*_X_*/Ag contact (hole injecting), in agreement with previous reports on similar device structures.^3,5^ The transmittance of the MoO*_X_*/Ag electrode is at around 50–60% in the emission wavelength range of SY, as shown in Figure S7. A schematic diagram of layer energy levels relevant to electroluminescence is presented in Figure 4b. An Al electrode injects the charges into the oxide layer. The injected electrons travel in the channel formed along the semiconductor/dielectric interface to be subsequently injected into the SY emissive layer. The presence of the ZnO NWs boosts this electron injection into the SY layer while also enhancing the outcoupling of the emitted light. The Cs_2_CO_3_ layer facilitates electron injection into the LUMO level of the SY layer by reducing the potential barrier between ZnO and SY. Further details can be found in the Experimental Section. The aspect ratio of the as-grown NWs also plays an important role, as evidenced by the clear differences in the performance of S-NW and L-NW HLETs, with the longer NWs having better overall operation. This is ascribed to the smaller diameter of the L-NWs (as discussed in Figure 1), which helps improve the injection of charges while lowering the contact resistance provided in Figure 4c using the transmission line method. We assume the main change in contact resistance is coming from the large area of NWs as the contact resistance drops with an increase in the size of NWs, and it promotes the hole injection.

To understand how the nanostructures assist light escaping from inside the device, we performed ray tracing simulations using the general-purpose photovoltaic device model (www.gpvdm.com).^28^ AFM height profiles (see Figure S8) were first taken from the ZnO nanostructures. These height profiles were then discretized using a regular 3D triangular mesh (see Figures S9 and S10). The mesh was then simplified using a vertex removal strategy and turned into a closed volume by adding a bottom and sides to it. This structure was then inserted into a bilayer simulation consisting of solid ZnO and Super Yellow. Ray tracing assumes light propagates as a particle and reflects and transmits at interfaces according to Snell’s law. The Fresnel equations were used to calculate reflection/transition coefficients. Refractive index values as a function of wavelength were obtained by using ellipsometry. Simulations were performed for the L-NWs, S-NWs, and control structures. Using this method, we were able to calculate the extraction efficiency of light emitted within the Super Yellow for the different measured nanostructures. The results can be seen in Figure 4d.

Further details on the escape efficiency of light as a function of the maximum height of the nanowires embedded in the Super Yellow are provided in the Supporting Information (see Figure S11). This was calculated by adjusting the measured height profile of the L-NWs in the simulation. Nanowire films with higher thicknesses enhance extraction efficiency between 525 and 625 nm, which is the wavelength range where Super Yellow emits, as shown in Figure 4a. Overall, the reduced contact resistance for injection of the holes (minority carriers) and improved outcoupling in the NW device resulted in an improved optical performance in the HLETs.

## Conclusions

In conclusion, we have demonstrated efficient solution-processed hybrid light-emitting transistors using ZnO NWs in combination with an emissive polymer in an asymmetric electrode device architecture. The presence of NWs does not significantly affect the electron mobility μ_e_ value in the range 0.6–0.25 cm^2^ V^–1^ s^–1^. However, it drastically reduces the ON/OFF ratio. Likewise, the incorporation of NWs into the transistor channel plays a critical role in the improvement of the optical performance of the HLETs. Importantly, the HLETs showed a high maximum EQE of ≈1.2% at luminance values in excess of 1000 cd m^–2^. The introduction of NWs has been shown to improve hole injection into the light-emissive Super Yellow layer, leading to more efficient radiative recombination. We also observed that NWs facilitate light outcoupling from HLET devices, leading to improved efficiency. It is evident that the introduction of NWs significantly enhances the optical performance of the HLETs. Although the operating voltages of the demonstrated HLETs are still high (40 V), these could be reduced by implementing lower channel length and increasing the gate capacitance by employing high k dielectrics or electrolyte gating.^29,30^ As such, our results present a significant performance advancement of solution-processed HLET toward numerous potential applications, including lighting, optical communication, smart display pixels, and integrated optoelectronic systems.

## Experimental Section

### NW Growth and Fabrication

The ZTO/ZnO active layer used in this paper was prepared by spin coating as a ZTO–ZnO layered stack. Prior to spin-coating oxide layers, the Si^++/^SiN_*x*_ substrates were thoroughly cleaned with acetone, isopropyl alcohol (IPA), and deionized water to remove any grease or residual dirt, dried with nitrogen, and subsequently treated in a UV-ozone environment for 15 min to ensure the correct wettability of the Si substrates. For the spin coating of the ZnO and ZTO layers, ZnCl_2_ (150 mM) and SnCl_2_ (150 mM) powders (anhydrous, 99.999% from Sigma-Aldrich) were dissolved in 2-methoxyethanol and stirred for 24 h to obtain a clear homogeneous solution. The ZTO solution was prepared from a 1:1 mixture of ZnCl_2_ and SnCl_2_ solutions. These precursor solutions were deposited on the Si substrates by spin coating at 5000 rpm for 60 s, followed by thermal treatment. The temperature of this thermal treatment depended on the layer of the stack. Hence, the ZTO and ZnO layers were treated for an hour at 400 and 300 °C, respectively. The overall three-layer stack (two layers of ZTO and one layer of ZnO) forming one active layer was prepared by spin coating and annealing each layer individually, with subsequent layers being prepared on top of the previous one. These conditions were selected as optimal for the electrical performance of the HLETs.

For the ZnO NW growth, an equimolar aqueous solution of zinc nitrate hexahydrate {Zn(NO_3_)·6H_2_O} and hexamethylenetetramine (HMTA, C_6_H_12_N_4_) was prepared, as per our previous work,^16^ with its concentration depending on the sample. After stirring the solids for 30 min, a clear solution was obtained. Then, the as-prepared Si substrates with the ZTO/ZnO active layer stack were introduced into the ZnO NW growth solution and placed face down on a custom-made sample holder. Then, the solution was introduced into an oven at 90 °C for 2–4 h, depending on the type of NWs produced. For S-NWs, we used a 25 mM equimolar solution that was placed in the oven for 2 h, while for the L-NWs, a 50 mM solution was prepared and subsequently placed in the oven for 4 h. These different ZnO NW growth conditions result in different dimensions of the studied NWs.

Super Yellow (SY, PDY-132) (see Figure S1a, for the chemical structure) was purchased from Merck. Super Yellow was dissolved in toluene at a 7 mg mL^–1^ concentration. An Al source electrode of 80 nm was deposited through a shadow mask in a high vacuum on top of the oxide layer in all three control, S-NW, and L-NW substrates. Following the same process, Cs_2_CO_3_ (8 nm) was deposited as the top contact, using the same shadow mask. Super Yellow (20 mg mL^–1^) was then spin-coated at 500 rpm for 30 s, followed by annealing at 150 °C for 30 min. The SY deposition and annealing were repeated the second time to cover the NWs in the channel completely. The thickness of SY was measured ∼350 nm on a flat surface. The device structure was completed by the deposition of the drain electrode consisting of MoO*_X_* (10 nm) and Ag (25 nm) by thermal deposition through a shadow mask on top of the SY layer resulting in an asymmetric contact configuration.^31^ The transistor channel length and width were 60–120 μm and 2 mm, respectively.

### Characterization and Measurements

The electrical characterization of the HLET devices was performed in a nitrogen-filled glovebox using two Agilent B2912A semiconductor parameter analyzers. Electroluminescence (EL) and photoluminescence (PL) spectra were measured using an optical fiber mounted above devices and connected to a USB spectrometer (Ocean-Optics USB4000-XR). Charge carrier mobility was calculated from the transistors’ transfer characteristics in the saturation regime. The luminance of the devices was determined from the photocurrent generated by a calibrated photodiode referenced to a standard luminance meter (Minolta LS-100), considering the relative emission area. EQE was obtained from the luminance, source-drain current (*I*_DS_), photocurrent and emission spectra of the devices, assuming Lambertian emission as reported in the past.^31,32^

AFM micrographs were obtained using a Bruker Multimode 8 scanning probe microscope with a Nanoscope V controller. Topography measurements were carried out using PeakForce QNM mode using NuNano Scout 350 probes with a nominal spring constant of 42 N/m. Images were captured with 512 × 512 line resolution and analyzed using Bruker NanoScope Analysis V1.5 software.

Absorption spectra of the MoOx/Ag layers deposited on quartz substrates were recorded with a UV-3600 double-beam spectrophotometer (Shimadzu).

Field emission scanning electron microscopy (FESEM) images of the ZnO NWs were obtained by using a FEI Helios Nanolab 600 at 15 kV. High-resolution transmission electron microscopy (HRTEM) and energy dispersive X-ray spectroscopy (EDX) were carried out in a JEOL 2100F FEG at 200 kV, equipped with an Oxford INCAx-sight Si (Li) detector with a 50 mm^2^ area at a 25° takeoff angle. The cross-sectional NW samples were prepared in the FESEM system, equipped with a Ga focused ion beam (FIB) source. Pt was deposited on the samples prior to FIB milling to ensure a smooth and clean cross-section of the NWs was obtained.

## Supporting Information Available

Detailed information on the molecular structure of emissive material, SEM and AFM micrographs of the ZTO layer and ZnO NWs; TEM micrographs of ZnO nanowires; HRTEM micrographs and SAED pattern of S-ZnO NWs; device structure; transmittance from the electrodes; 3D reconstruction of AFM images in GPVDM; modeling the ray tracing simulation; calculations of efficiency as a function of layer height of the L-NWs layer, and a comparison with the literature (PDF)
