# Supplementary material for: Nanostructured Channel for Improving Emission Efficiency of Hybrid Light-Emitting Field-Effect Transistors
Source: ACS Photonics. 2023 Dec 10;10(12):4315–21. doi: 10.1021/acsphotonics.3c01080 (PMC10739997; doi:10.1021/acsphotonics.3c01080)
Supplement: Supplementary file 1 — ph3c01080_si_001.pdf [file ph3c01080_si_001.pdf]

Supporting Information

Nanostructured channel for improving emission efficiency of hybrid light-emitting field-effect transistors

Alejandro Galán-González*[1,2], Piotr Pander[3,4], Roderick C. I. MacKenzie[1], Leon Bowen[5], Dagou A. Zeze[1], Robert J. Borthwick[1], Richard L. Thompson[6], Fernando B. Dias [5], Mujeeb Ullah Chaudhry*[1]

[1] Department of Engineering, Durham University, Durham, DH1 3LE, United Kingdom

[2] Instituto de Carboquímica (ICB-CSIC), C/ Miguel Luesma Castán 4, 50018, Zaragoza, Spain

[3] Faculty of Chemistry, Silesian University of Technology, Strzody 9, 44-100 Gliwice, Poland

[4] Centre for Organic and Nanohybrid Electronics, Silesian University of Technology, Konarskiego 22B, 44-100 Gliwice, Poland

[5] Department of Physics, Durham University, Durham, DH1 3LE, United Kingdom

[6] Department of Chemistry, Durham University, Durham, DH1 3LE, United Kingdom

*Corresponding Authors: alejandro.galan@udc.es ; mujeeb.u.chaudhry@durham.ac.uk

List of Figures:

Figure S1. Molecular structure of SY, SEM and AFM micrographs of the ZTO layer.

Figure S2. HRTEM micrograph and SAED pattern of S-ZnO NWs.

Figure S3. TEM micrographs of ZnO nanowires.

Figure S4. Gate leakage current in control, S-NWs, and L-NWs LEEFTs.

Figure S5. Output characteristics of control, S-NWs, and L-NWs LEEFTs.

Figure S6. Variable channel HLET image.

Figure S7. Transmittance from the top MoOx/Ag electrodes.

Figure S8. AFM images of NWs.

Figure S9. 3D reconstruction of AFM images in GPVDM.

Figure S10. Ray tracing simulation example 300-700nm.

Figure S11. Calculation efficiency as a function of layer height of the L-NWs layer.

Table 1. Comparison with literature

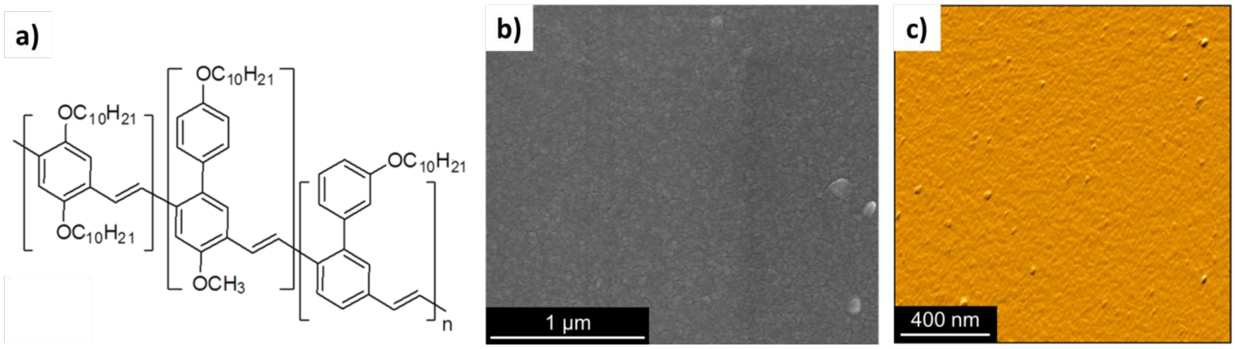

Figure S1. a) Molecular Structure of SY (PDY-132), b) SEM and, c) AFM micrographs of the ZTO layer.

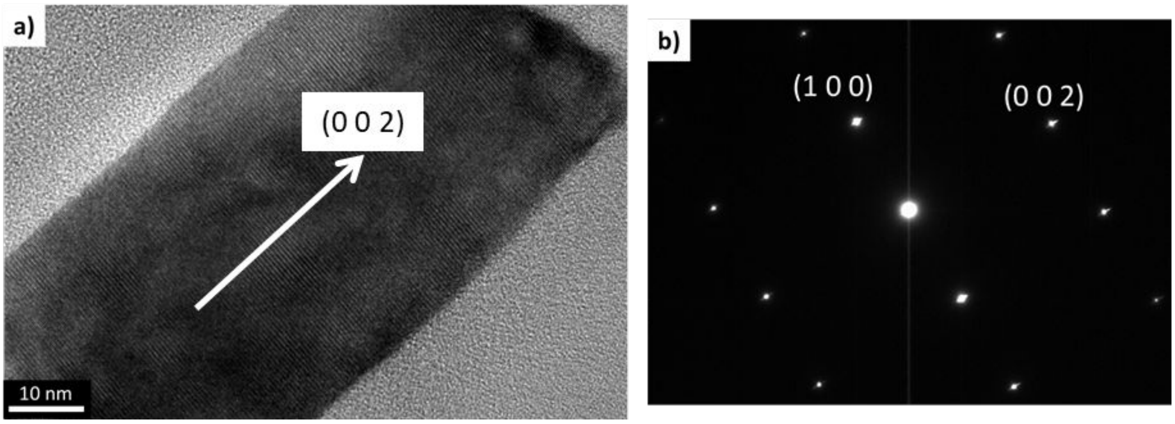

Figure S2. a) HRTEM micrograph and b) SAED pattern of the S-NWs, showing their clear single crystal quality. The nanowires growth direction is the (0 0 2), hence indicating the wurtzite crystalline structure [1].

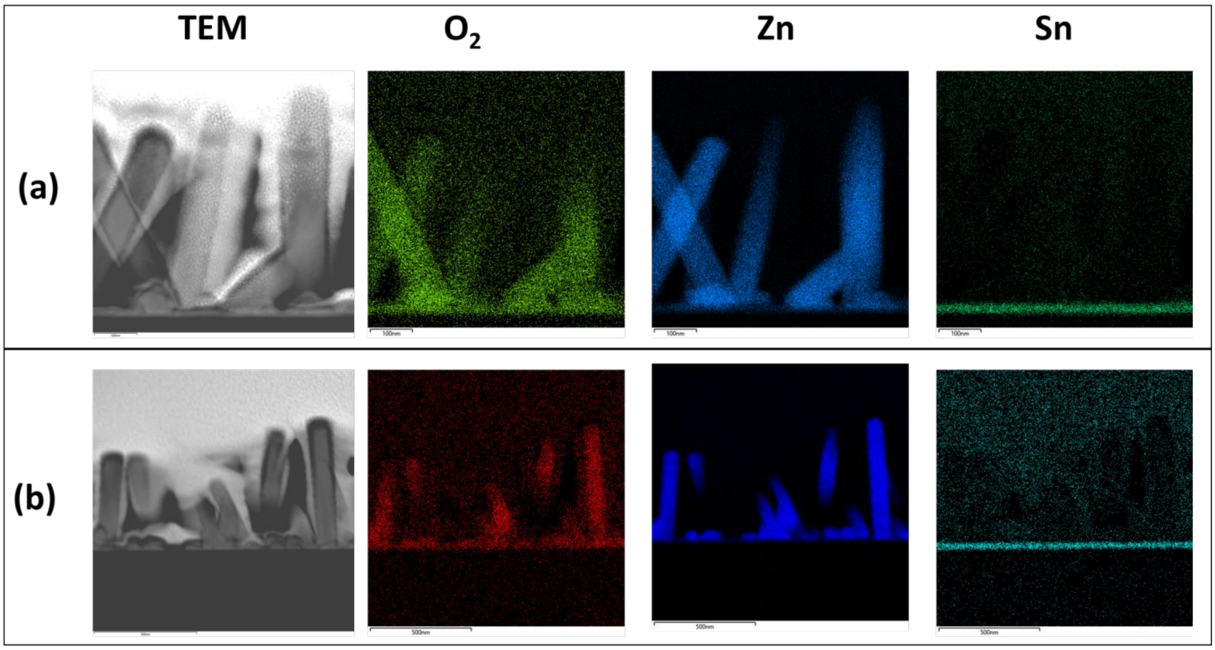

Figure S3. TEM micrographs of the a) short, and b) long ZnO nanowires that cap the channel ZTO layer. The O_2, Zn and Sn columns show the STEM elemental mapping carried out on the TEM micrographs. This clearly demonstrates that the nanowires are solely formed by ZnO and that the Sn is constrained to the ZTO/ZnO stack.

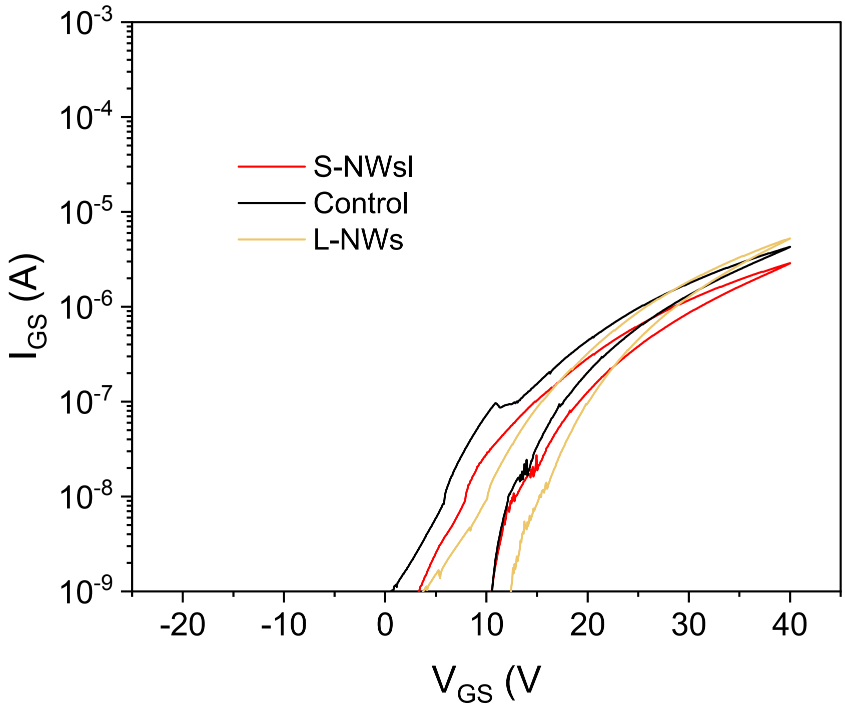

Figure S4. Gate leakage current in control, S-NWs, and L-NWs LEEFTs.

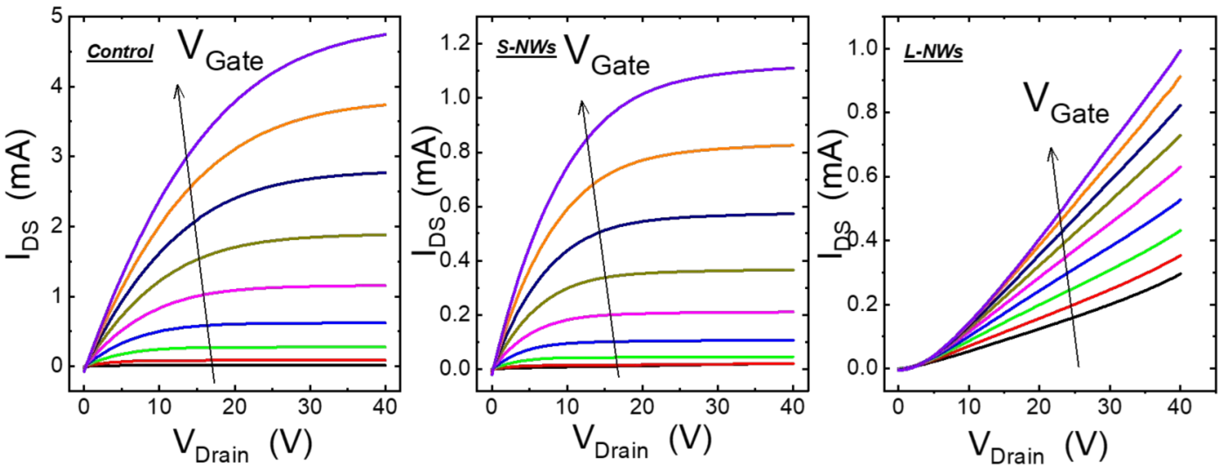

Figure S5. Output characteristics of control, S-NWs, and L-NWs LEEFTs.

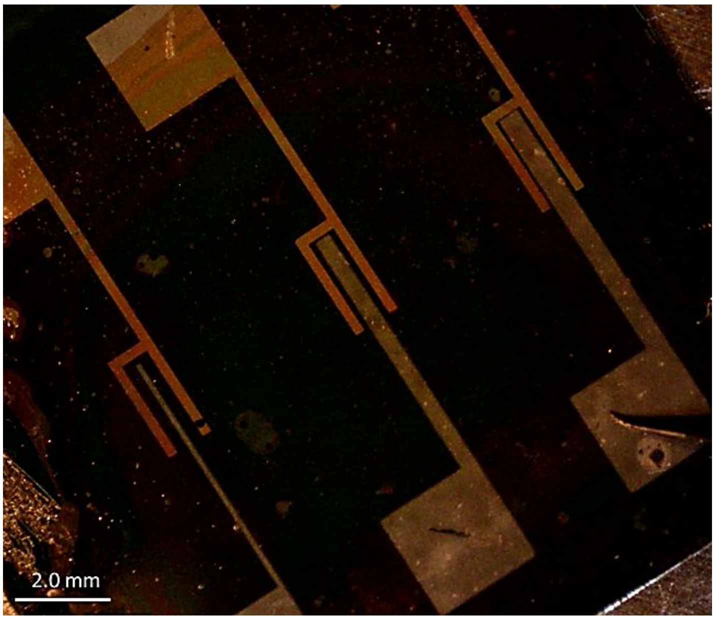

Figure S6. Image of the HLETs with the variable channel lengths used to calculate total and contact resistance.

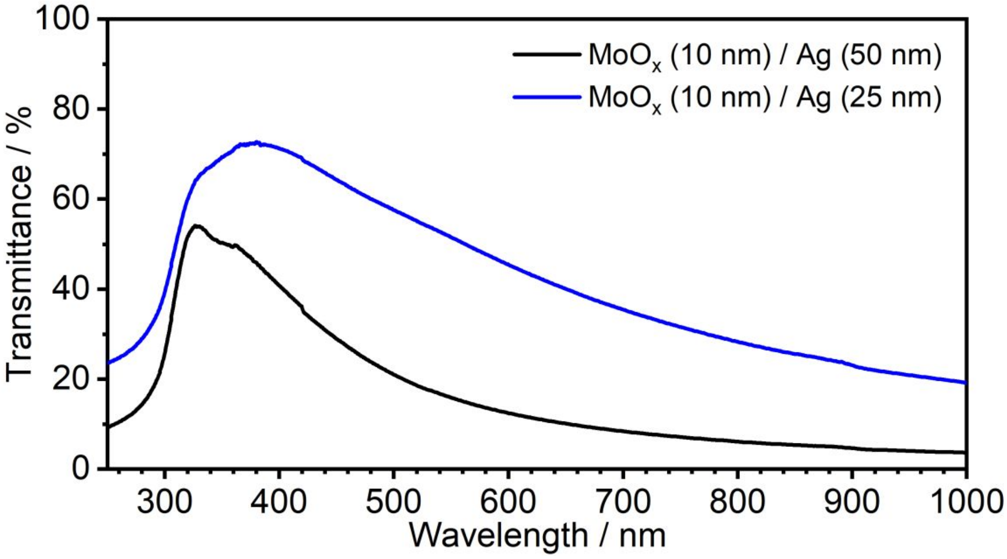

Figure S7. The transmittance of the top MoOx/Ag electrode with two thicknesses of the Ag layer.

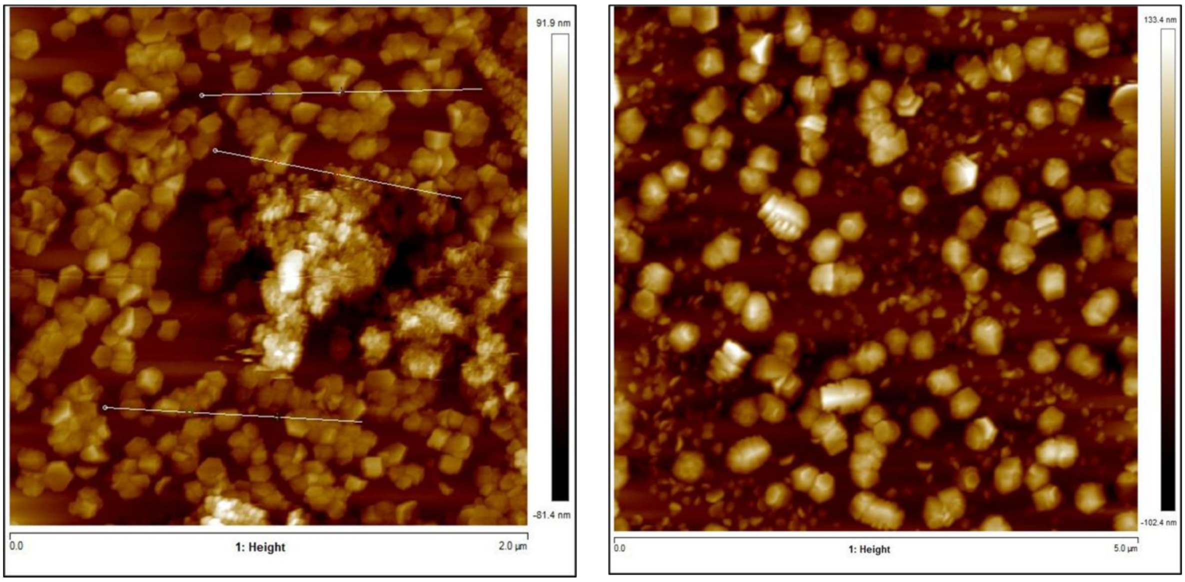

Figure S8. AFM height profiles of the ZnO nanostructures.

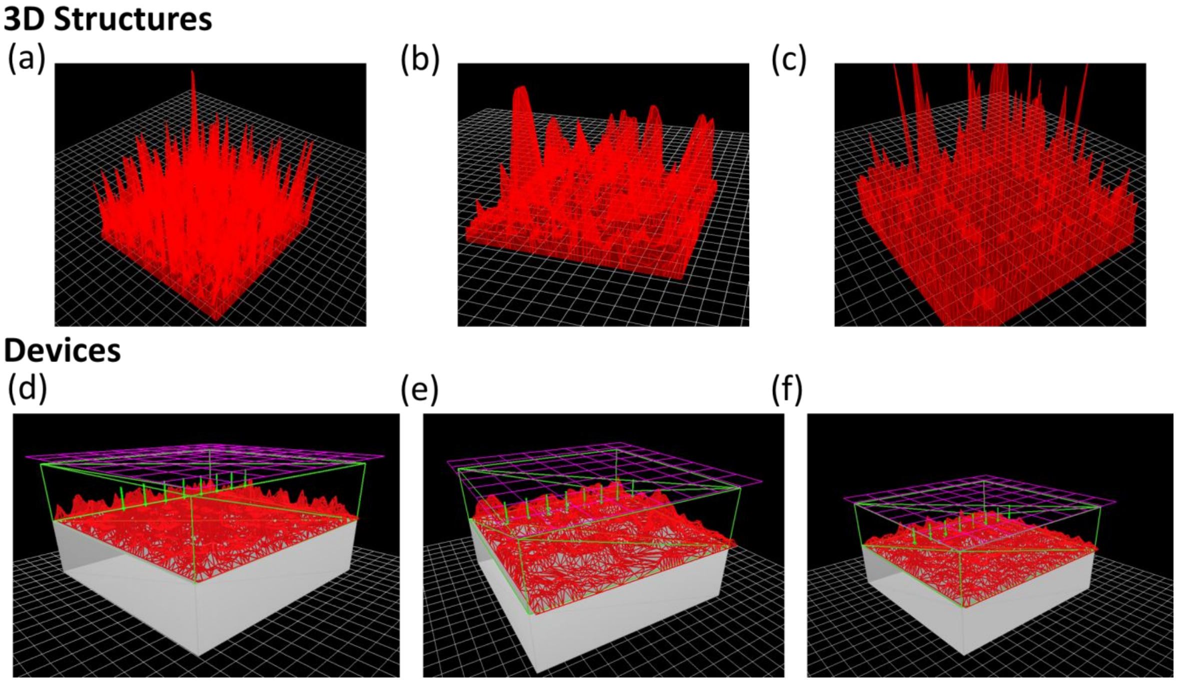

Figure S9. 3D reconstruction of AFM images and devices in GPVDM.

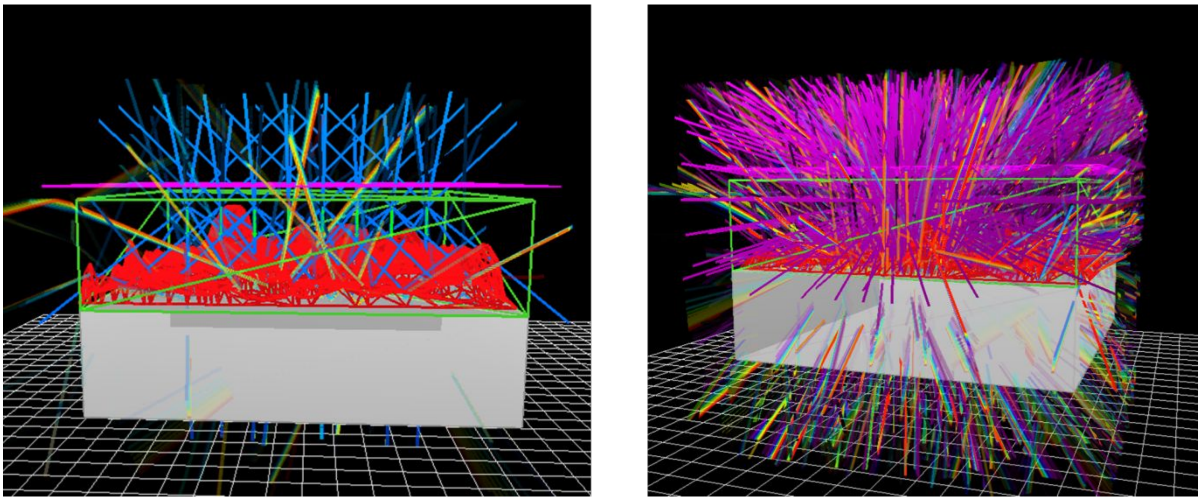

Figure S10. Ray tracing simulation example for the wavelength range 300-700nm.

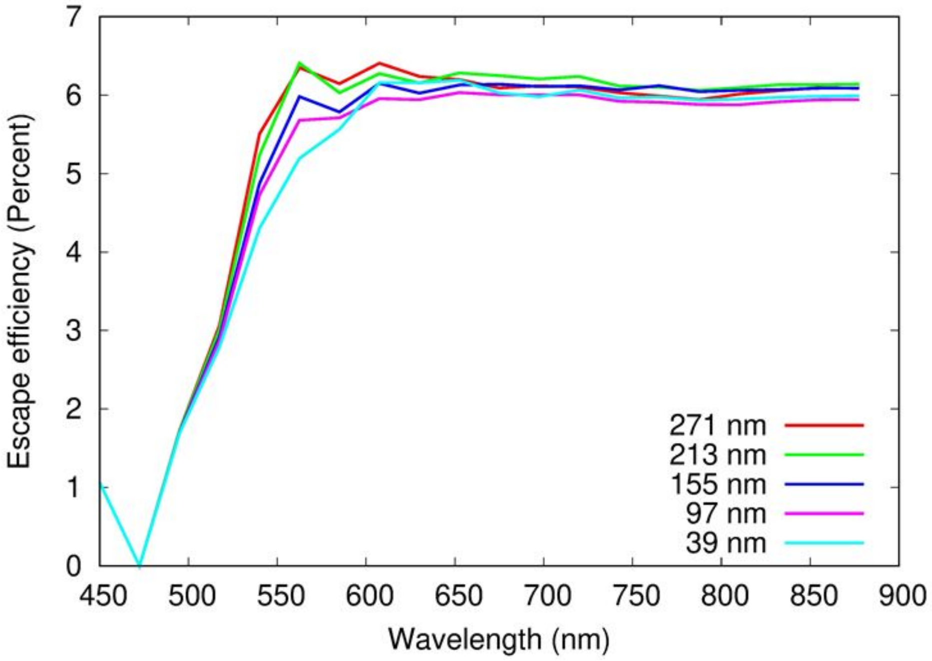

Figure S11. Calculation of the escape efficiency as a function of layer height of the L-NWs layer.

Table S1. Comparison of the HLET performance of this work with the literature

**Reference**	This study	Muhieddine et al [2]	Muhieddine et al [3]	Ullah et al [4]	Yamada et al [5]	Ooi et al [6]	Nakanotani et al [7]
**Oxide**	Solution Processed ZnO NWs on ZTO	Solution Processed ZTO	Solution Processed ZTO	Solution Processed IGZO	Solution Processed ZTO	Solution Processed ZTO	Sputtered IZO
**Mobility** ***$cm^2\ V^{-1}\ s^{-1}$***	0.5	4.2	1.62	22	8.1×10^{-1}	4.2	13.8
**Luminance** ***$cd\ m^{-2}$***	1250	413	1330	1800	Not Reported	413	Not Reported
**EQE %**	1.2	0.0018	0.087	0.5	Not Reported	0.0018	6.7×10^{-5}

References for Supporting Information:

1. Galán-González, A.; Sivan, A. K.; Hernández-Ferrer, J.; Bowen, L.; Di Mario, L.; Martelli, F.; Benito, A. M.; Maser, W. K.; Ullah, M.; Gallant, G.; Zeze, D. A.; Atkinson, D. Cobalt-Doped ZnO Nanorods Coated with Nanoscale Metal–Organic Framework Shells for Water-Splitting Photoanodes. *ACS Appl. Nano Mater.* **2020**, 3, 8, 7781–7788.

2. Muhieddine, K.; Ullah, M.; Pal, B. N.; Burn, P.; Namdas, E. B. All Solution-Processed, Hybrid Light Emitting Field-Effect Transistors. Adv. Mater. **2014**, 26, 37, 6410.

3. Muhieddine, K;. Ullah, M.; Maasoumi, F.; Burn, P. L.; Namdas, E. B. Hybrid Area Emitting Transistors: Solution Processable and with High Aperture Ratios. Adv. Mater. **2015**, 27, 42, 6677.

4. Ullah, M et al. Hybrid Light-Emitting Transistors Based on Low-Temperature Solution-Processed Metal Oxides and a Charge-Injecting Interlayer. Adv. Opt. Mater. **2016**, 4,02, 231.

5. Yamada, K; Yamao, T.; Hotta, S. Light-Emitting Field-Effect Transistors Having Combined Organic Semiconductor and Metal Oxide Layers. Adv. Mater. **2013**, 25, pp. 2860- 2866.

6. E. Ooi et al. A Light Emitting Transistor Based on a Hybrid Metal Oxide-Organic Semiconductor Lateral Heterostructure, Z. Appl. Phys. Lett. **2012**, 100, 093302.

7. Nakanotani, H.; Yahiro, M.; Adachi, C; Yano, K. Ambipolar Field-Effect Transistor Based on

Organic-Inorganic Hybrid Structure. Appl. Phys. Lett. **2007**, 90, 262104
